# Role of the Two Component Signal Transduction System CpxAR in Conferring Cefepime and Chloramphenicol Resistance in *Klebsiella pneumoniae* NTUH-K2044

**DOI:** 10.1371/journal.pone.0033777

**Published:** 2012-04-04

**Authors:** Vijaya Bharathi Srinivasan, Vasanth Vaidyanathan, Amitabha Mondal, Govindan Rajamohan

**Affiliations:** Council of Scientific Industrial Research - Institute of Microbial Technology, Sector 39 A, Chandigarh, India; Université d'Auvergne Clermont 1, France

## Abstract

**Background:**

*Klebsiella pneumoniae* is a Gram-negative, non-motile, facultative anaerobe belonging to the Enterobacteriaceae family of the γ-Proteobacteria class in the phylum Proteobacteria. Multidrug resistant *K. pneumoniae* have caused major therapeutic problems worldwide due to emergence of extended-spectrum β-lactamase producing strains. Two-component systems serve as a basic stimulus-response coupling mechanism to allow organisms to sense and respond to changes in many different environmental conditions including antibiotic stress.

**Principal Findings:**

In the present study, we investigated the role of an uncharacterized *cpxAR* operon in bacterial physiology and antimicrobial resistance by generating isogenic mutant (Δ*cpxAR*) deficient in the CpxA/CpxR component derived from the hyper mucoidal K1 strain *K. pneumoniae* NTUH-K2044. The behaviour of Δ*cpxAR* was determined under hostile conditions, reproducing stresses encountered in the gastrointestinal environment and deletion resulted in higher sensitivity to bile, osmotic and acid stresses. The Δ*cpxAR* was more susceptible to β-lactams and chloramphenicol than the wild-type strain, and complementation restored the altered phenotypes. The relative change in expression of *acrB, acrD, eefB* efflux genes were decreased in *cpxAR* mutant as evidenced by qRT-PCR. Comparison of outer membrane protein profiles indicated a conspicuous difference in the knock out background. Gel shift assays demonstrated direct binding of CpxR^KP^ to promoter region of *ompC* ^KP^ in a concentration dependent manner.

**Conclusions and Significance:**

The Cpx envelope stress response system is known to be activated by alterations in pH, membrane composition and misfolded proteins, and this systematic investigation reveals its direct involvement in conferring antimicrobial resistance against clinically significant antibiotics for the very first time. Overall results displayed in this report reflect the pleiotropic role of the CpxAR signaling system and diversity of the antibiotic resistome in hyper virulent K1 serotype *K. pneumoniae* NTUH-K2044.

## Introduction

Bacteria can encounter numerous environments in which chemical and physical factors such as osmotic pressure, temperature, pH and carbon source availability can change considerably and unpredictably [1]. To adapt to changing conditions, bacteria possess an array of mechanisms which sense external factors and respond accordingly, central to this are the two component systems (TCS) [2].

TCS are signal transduction devices found in all domains of life, and they are especially widespread in bacteria. These systems regulate diverse responses, including nutrient acquisition, energy metabolism, adapting to environmental cues, complex developmental pathways, and host-pathogen interactions. TCS are typically composed of a transmembrane sensor Histidine Kinase protein (HK) and a cytoplasmic transcriptional Response Regulator (RR) [3]. The transmembrane sensor component harbors at least two domains: an input domain that senses the environmental stimulus and a cytoplasmic transmitter with kinase activity that alters the external stimulus into an adaptive signal by autophosphorylation at a conserved histidine residue. The phosphorylated histidine is the resource for phosphorylation of a conserved aspartic acid residue in the receiver domain of the RR. The phosphorylated RR then mediates the cellular response, usually by differential expression of target genes. The target genes of a particular TCS are customized to the specific signal to which the particular TCS corresponds. This specificity is also reflected by the high specificity of HK and RR pairs [3].

The Cpx envelope stress response is controlled by the TCS consisting of membrane localized sensor kinase CpxA and the regulator CpxR. CpxA is induced by a variety of envelope stresses, all of which are predicted to result in protein misfolding. These include physical (osmolarity), chemical (ethanol, pH, indole), and biological (adhesion, lipids) stresses, misfolded proteins (adhesin subunits, β-barrel outer membrane proteins, and misfolded variants of the maltose-binding protein), copper, detergents, and EDTA.

Activation of CpxA involves relief of inhibition through proteolysis of the periplasmic protein CpxP, in addition to other events that require the periplasmic sensing domain of CpxA [4]. This leads to a cascade of phosphotransfer events that ultimately causes a build up of phosphorylated CpxR (CpxR∼P), which functions as a transcription factor to activate and, in a small number of cases, repress transcription of target genes. A set of the target genes encodes envelope protein folding and degrading factors, such as the periplasmic protease/chaperone DegP/HtrA, the major disulphide oxidase DsbA and two peptidyl-prolyl-isomerases PpiA and PpiD [5]. Accordingly, a major role of the Cpx response appears to be to maintain envelope protein folding status in the presence of adverse conditions. Molecular, biological and biochemical analysis of several Cpx signals supports the notion that most signals are specific. Therefore the Cpx system serves as an efficient model system to determine the mechanisms involved in signal transduction by a TCS, ranging from signal integration by the kinase CpxA to the output response by CpxR [6].

Studies have elucidated the different functions displayed by the Cpx system for example in envelope stress response system, pilus assembly, type III secretion, motility and chemotaxis, adherence, and biofilm development [6]. Furthermore, the Cpx system is required for invasion of host cells in diverse pathogenic bacteria, including *Escherichia coli*, *Salmonella enterica* serovar Typhimurium, *Shigella sonnei*, *Yersinia enterocolitica*, and *Legionella pneumophila* [6]–[7]. A recent study demonstrated that *Xenorhabdus nematophilus* requires the Cpx system both to colonize its nematode host (*Steinernema carpocapsae*) and to kill larvae of the tobacco hornworm (*Manduca sexta*) [8]. Overall, the Cpx system of bacteria is known to be involved in maintenance, adapt and protection of the bacterial envelope in response to a variety of stressors, one such environmental assail that pathogenic bacteria for example *E. coli, Salmonella spp., Enterobacter spp., Campylobacter spp., Acinetobacter spp., Pseudomonas spp.,* including *Klebsiella spp.,* get exposed to is the pressure of antibiotics. Though the Cpx system has been implicated in the multidrug resistance (MDR) of various human pathogens, however its direct involvement in regulating antimicrobial resistance has remained completely unexplored. Here, we initiated a systematic study to elucidate the direct role of Cpx TCS in conferring drug resistance by constructing a *cpxAR* deletion mutant in a notoriously drug resistant model organism; *Klebsiella pneumoniae*.

*K. pneumoniae* are opportunistic pathogens and can give rise to severe diseases such as septicemia, pneumonia, urinary tract infections, and soft tissue infection. The hospitalized, immune compromised patient with underlying diseases is the main target of these bacteria. Thus, *Klebsiella* infections may serve as a paradigm of hospital-acquired infections. Their incidence of 5 to 7% of all hospital-acquired infections ranks them among the most important nosocomial pathogens. *Klebsiella* infections are not only responsible for nosocomial infections but also for community acquired infections such as severe liver abscesses. The capsular polysaccharide on their surface is the prime factor of virulence and toxicity in causing pyogenic liver abscess, with a high 10–30% mortality rate globally [9]. The *K. pneumoniae* NTUH-K2044 strain encapsulated with K1 hyper virulent antigen is usually isolated from clinical liver abscess patients. Increasingly, *Klebsiella* bacteria have developed antibiotic resistance, most recently to the class of antibiotics known as carbapenems. The MisT2 database (http://mistdb.com/) has shown the presence of >466 signaling proteins in the 5,472,672 bp (GC content: 57.4%) genome sequence of the K1 serotype (Accession No: AP006725.1) [10]. Though the biological functions of few TCS have been demonstrated previously; however the role of Cpx system has never been examined. Due to their central role in bacterial virulence regulation and their absence in animals including humans, TCS have been suggested as targets for antimicrobials, thus it is prudent to investigate their role in bacterial physiology in general and drug resistance in particular.

In this report experimental evidence for the various physiological functions displayed by CpxAR system in *K. pneumoniae* NTUH-K2044 has been demonstrated for the very first time.

## Results

### Bioinformatic analysis of KP1_0078 (*cpxA*)/KP1_0079 (*cpxR*) TCS

**Figure 1:**
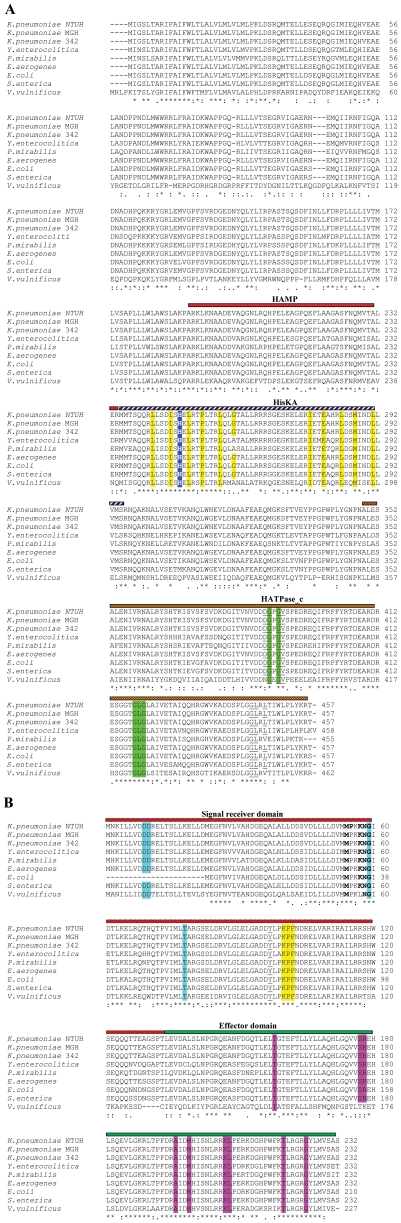
Multiple sequence alignment of KP1_0078 (CpxA homolog) and KP1_0079 (CpxR homolog) from other Gram negative bacteria. **A**)Sequence alignment of KP1_0078 with CpxA from other bacteria. Sequence based multiple alignment of KP1_0078 with CpxA homologues. The accession numbers (given in brackets) of CpxR from different bacteria; *K. pneumoniae* NTUH-K2044 (BAH60994.1), *K. pneumoniae* MGH78578 (ABR79598.1), *K. pneumoniae* 342 (YP_002241207.1), *Y. enterocolitica* (YP_001004479.1), *P. mirabilis* (YP_002152884.1), *E. aerogenes* (YP_004591708.1), *E. coli* (YP_003231578.1), *S.* Typhimurium (NP_462939.1), *V. vulnificus* (NP_935907.1). Clustal W alignment (www.ebi.ac.uk/clustalw) of CpxA illustrates three conserved domains such as HAMP (amino acids 191–234), HisKA (amino acids 236–296) and HATPase_ c (amino acids 350–451). Probable phosphorylation site which are conserved are shown in blue shaded boxes with bold letters and dimer interface residues are yellow shaded. Residues that are conserved in ATP binding including the G-X-G motifs are highlighted in green and underlined. B) Sequence alignment of KP1_0079 with CpxR from other bacteria. Sequence based multiple alignment of KP1_0079 with CpxR homologues. The accession numbers (given in brackets) of CpxR from different bacteria; *K. pneumoniae* NTUH-K2044 (YP_002917062.1), MGH78578 (YP_001337866.1), *K. pneumoniae* 342 (YP_002241206.1), *Y. enterocolitica* (YP_001004480.1), *P. mirabilis* (YP_002152885.1), *E. aerogenes* (YP_004591709.1), *E. coli* (EFZ74944.1), *S.* Typhimurium (NP_462940.1), *V. vulnificus* (NP_760183.1). ClustalW alignment of CpxR revealed conserved two domains encompassing signal receiver (amino acids 5–115) and effector domain (amino acids 134–228). The conserved domain analyses (www.ncbi.nlm.nih.gov/Structure/cdd/cdd.shtml) were performed to identify the functionally important residues. Conserved residues contributing to the formation of active site are shown in light blue shade and probable intermolecular recognition sites which are close to active site are highlighted in bold. Probable DNA binding residues are highlighted in magenta. (*) represents identical residues, (:) and (.) indicates high and low similarity respectively.

The *K. pneumoniae* gene KP1_0078 (1374 bp, 457aa) upon performing BLAST exhibits >75% identity (in brackets) with CpxA protein of other organisms with the closest homolog being CpxA of *Enterobacter aerogenes* (99%), *S.* Typhimurium (96%), *E. coli* (95%), *Shigella flexneri* (95%), *Proteus mirabilis* (86%), *Yersinia pestis* (88%) [4], [6]. Structural motifs present in *K. pneumoniae* CpxA mark it as the sensor/HK component of a TCS. Secondary-structure prediction and comparison to known HK structures indicates that this CpxA has a short, cytoplasmic part (amino acids 1 to 8), followed by a transmembrane helix (amino acids 9 to 27), residues 28 to 164 comprise the extracellular region with mixed secondary structures and probably which serve as the sensor, residues 165 to 182 forms the second transmembrane helix and amino acids from 183 to 457 comprises a large cytoplasmic region and other conserved residues/domains are present in its primary sequence (Figure 1A).

The KP1_0079 (699 bp, 232aa) gene encodes a 23 kDa protein with homology to CpxR proteins which are the RRs in TCSs. KP1_0079 exhibits highest level of homology to CpxR of *E. aerogenes* (99%), *S.* Typhimurium (96%), *E. coli* (95%), *S. flexneri* (95%), *P. mirabilis* (79%), and *Y. pestis* (81%) [4], [6]. Comparing *K. pneumoniae* CpxR to other RRs indicates that this CpxR has two domains: an N-terminal signal receiver domain (amino acids 5 to 115) and a C-terminal effector domain (amino acids 134 to 228) (Figure 1B). In the receiver domain, the residue (D51) necessary for phosphoryl transfer from CpxA is conserved.

An analysis of the primary sequence and the secondary-structure prediction for *K. pneumoniae* aligned CpxR to the sequence of the receiver domain of the structurally characterized YycF from *Bacillus subtilis* (Protein Data Bank accession number 2ZWM) which indicates that this domain is 50% identical to that of *K. pneumoniae* CpxR, suggesting that the structures of the two proteins are likely to be very similar. The C-terminal domain of *K. pneumoniae* CpxR very likely binds to DNA, given its sequence homology to other effector domains that have this function. A multiple sequence alignment of the proteins clearly showed that they were highly conserved throughout bacterial kingdom. Overall, *in silico* analysis suggests that KP1_0078/KP1_0079 is indeed a CpxA/CpxR signaling system worthy to be characterized.

### Construction and morphological analysis of *cpxAR* deletion mutant

The nucleotide sequence deduced from the 2073 bp DNA fragment obtained from *K. pneumoniae* NTUH-K2044 shared >75% identity with the CpxA/CpxR regulatory system of Gram negative pathogens. To determine the role of *cpxAR*, a *cpxAR* mutant was created by conjugation in the wild-type *K. pneumoniae* NTUH-K2044. This strain was selected due to its high virulence in a murine model of pneumonia [10]. We used insertion-duplication mutagenesis to interrupt *cpxAR*, required for the synthesis of a functional signaling system. PCR followed by DNA sequencing was done to confirm the disruption of the operon in *K. pneumoniae*. RT-PCR analysis confirmed that the mutations abolished the transcription of *cpxA* and *cpxR* (data not shown).

The *cpxAR* mutant appeared as smaller colonies (5.0–5.5 mm on average) compared to wild-type (6.0–6.5 mm on average), [wild type/Δ*cpxAR* mutant, P = 0.037669].

The average lengths of strings generated by NTUH-K2044 and NTUH-K2044Δ*cpxAR* mutant as per hypermucoviscosity string test were 5.12, 5.08 cm respectively.

The precipitation test was carried out on the 12 h grown culture in LB broth at 37°C. Both NTUH-K2044 and NTUH-K2044Δ*cpxAR* did not form a dense pellet after centrifuging at 10,000 g for 10 min.

**Figure 2:**
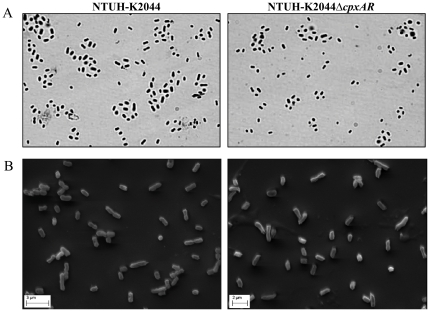
Characterization of Δ*cpxAR* mutant. **A**) Anthony's capsule staining was performed to visualize wild type and mutant in Olympus microscope work station. No difference was observed between wild type and mutant. B) The morphology of NTUH-K2044 (panel A) and NTUH-K2044Δ*cpxAR* (panel B) was determined by scanning electron microscopy. No phenotypic alteration was observed.

Visualization of cultures under microscope using 20% CuSO_4_ by Anthony's capsule staining methodology revealed no difference in the exopolysaccharide production around NTUH-K2044 and NTUH-K2044Δ*cpxAR* (Figure 2A). Quantification of glucuronic acid content reconfirmed the same observation (Table 1).

**Table 1:** Determination of capsular polysaccharides.

Strain	Glucuronic acid content (µg/10^9^ CFU)^a^	Mucoviscosity^b^
NTUH-K2044	17.29±1.21	Very strong
NTUH-K2044Δ*cpxAR*	16.88±2.87	Very strong
NTUH-K2044Δ*cpxARΏcpxAR*	16.98±2.53	Very strong

a Values are the averages of triplicate samples represented by mean ± standard deviation.

b Confirmed by string test.

The effect of deleting *cpxAR* on the colony morphology of *K. pneumoniae* was evaluated by scanning electron microscopy. The results indicated no significant difference in cell size of NTUH-K2044 (1.163 µm±0.4) and NTUH-K2044Δ*cpxAR* (1.196 µm±0.32) (Figure 2B).

### Role of *cpxAR* in bacterial growth

**Figure 3:**
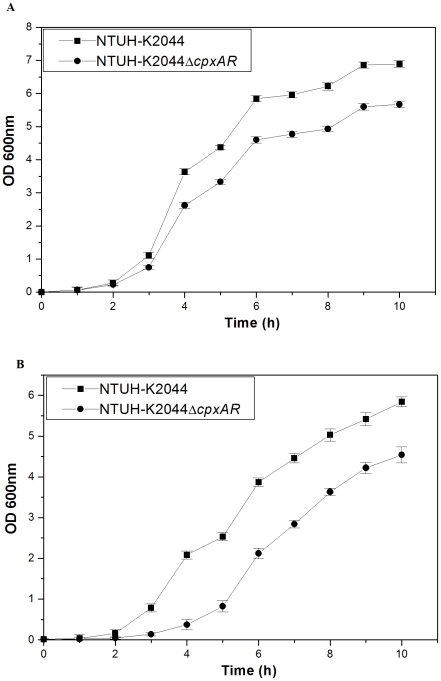
Mutant strain displayed stunted growth in LB at varying pH. Growth kinetics of NTUH-K2044, and NTUH-K2044Δ*cpxAR* was assessed in LB medium pH 6.0 (A) and pH 8.0 (B).

To decipher the involvement of Cpx signal transduction system in inducing a general or global response, the growth kinetics of Δ*cpxAR* strain was compared with that of the wild type strain. Experimentally the growth characteristics of NTUH-K2044 and NTUH-K2044Δ*cpxAR* were determined over a period of ∼10 h in LB medium with different pH and analysis revealed unique patterns. We tested the growth kinetics at pH 3.0, 6.0, 8.0 and 12.0 respectively. It was interesting to note that mutant exhibited 1.2–1.3 fold (±0.34) reduced growth compared to the wild type strain in LB at pH 6.0 (Figure 3A) [wild type/Δ*cpxAR* mutant, P = 0.000859].

The NTUH-K2044Δ*cpxAR* exhibited 5.6 fold stunted growth compared to NTUH-K2044 after 4 h, and displayed a 1.2 fold difference thereafter in LB at pH 8.0 (Figure 3B) [wild type/Δ*cpxAR* mutant, P = 0.000672]. The differences observed in growth profiles at pH6.0 and pH8.0 were not statistically different.

The other tested pH 3.0 and 12.0 was toxic to both the cultures. The apparent density of the Δ*cpxAR* culture was 1.3 fold (±0.59) lower compared to wild-type parent strain after 8 h.

### Role of *cpxAR* in bile and osmotic stress response

**Figure 4:**
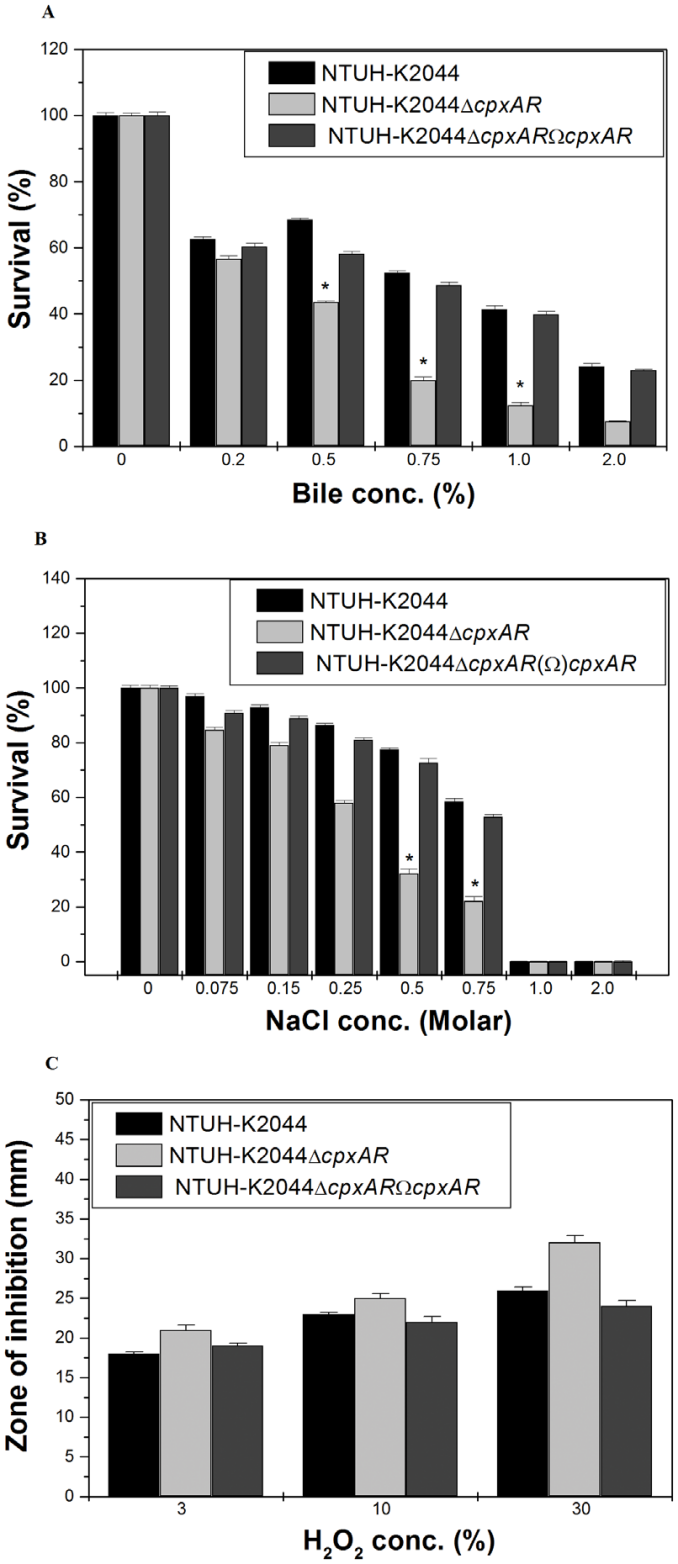
Survival of NTUH-K2044, NTUH-K2044Δ*cpxAR*and NTUH-K2044Δ*cpxAR*Ω*cpxAR* under different concentration of bile, osmotic and oxidative stress challenge A) Stress sensitivity of the NTUH-K2044 wild-type strain, the *cpxAR* mutant. The percentage of resistance to bile (0.2%, 0.5%, 0.75%, 1.0%, and 2.0%) was calculated by comparison to the numbers of viable cells in LB medium alone. B) The percentage of resistance to different concentration of osmotic stress (0.075 M, 0.15 M, 0.25 M, 0.5 M, 0.75 M, 1.0 M and 2.0 M) for NTUH-K2044, and NTUH-K2044Δ*cpxAR* was calculated by comparison to the numbers of viable cells in control. C) Oxidative stress response of Δ*cpxAR* mutant. The ability of NTUH-K2044, NTUH-K2044Δ *cpxAR* to combat different levels of hydrogen peroxide stress (3%, 10% and 30%) was measured by disc diffusion assay. The datas are the means of measurements made in triplicate and performed three times. *, Significant difference (P<0.05, Student t test).

To determine the role of *cpxAR* in intestinal colonization, NTUH-K2044 and NTUH-K2044Δ *cpxAR* underwent specific gastrointestinal (GI) stress associated with bile and osmotic challenges. In the bile resistance assay, NTUH-K2044 and mutant were exposed to different concentrations (physiological concentration is 0.2% to 2%, [11]) of bile. The ability of NTUH-K2044 to grow in the presence of 0.75% bile was 2.6 fold (±0.089), 1% was 3.5 fold (±0.059) and 2% was 3.2 fold (±0.04) higher when compared to NTUH-K2044Δ*cpxAR*, while transcomplemented NTUH-K2044Δ*cpxAR*Ω*cpxAR* strain restored the ability to tolerate stress (Figure 4A) [wild type/Δ*cpxAR* mutant, P = 0.0191; wild type/transcomplememted, P = 0.087].

The ability of NTUH-K2044 to grow in the presence of NaCl (physiological concentration being 150 mM, [12]) at 0.25 M was 1.5 fold (±0.024), 0.5 M was 2.5 fold (±0.033), and 0.75 M was 2.7 fold (±0.24), higher when compared to NTUH-K2044Δ*cpxAR* regardless of the inoculum size (Figure 4B) [wild type/Δ*cpxAR* mutant, P = 0.030051; wild type/transcomplememted, P = 0.012].

### Involvement of *cpxAR* in oxidative stress tolerance

To deduce whether *cpxAR* is a peroxide sensor and transcription regulator, we performed the hydrogen peroxide challenge assay. Disc diffusion assay showed that the *cpxAR* mutant had 1.2 fold greater sensitivity to 30% H_2_O_2_ (inhibition zone = 32±1.0 mm) than the wild-type (inhibition zone = 24±0.0 mm) (Figure 4C) [wild type/Δ*cpxAR* mutant, P = 0.0927; wild type/transcomplememted, P = 0.528], thereby demonstrating that the response of *K. pneumoniae cpxAR* mutant is conserved in oxidative stress.

### Association of *cpxAR* in antibiotic resistance in *K. pneumoniae*

**Figure 5:**
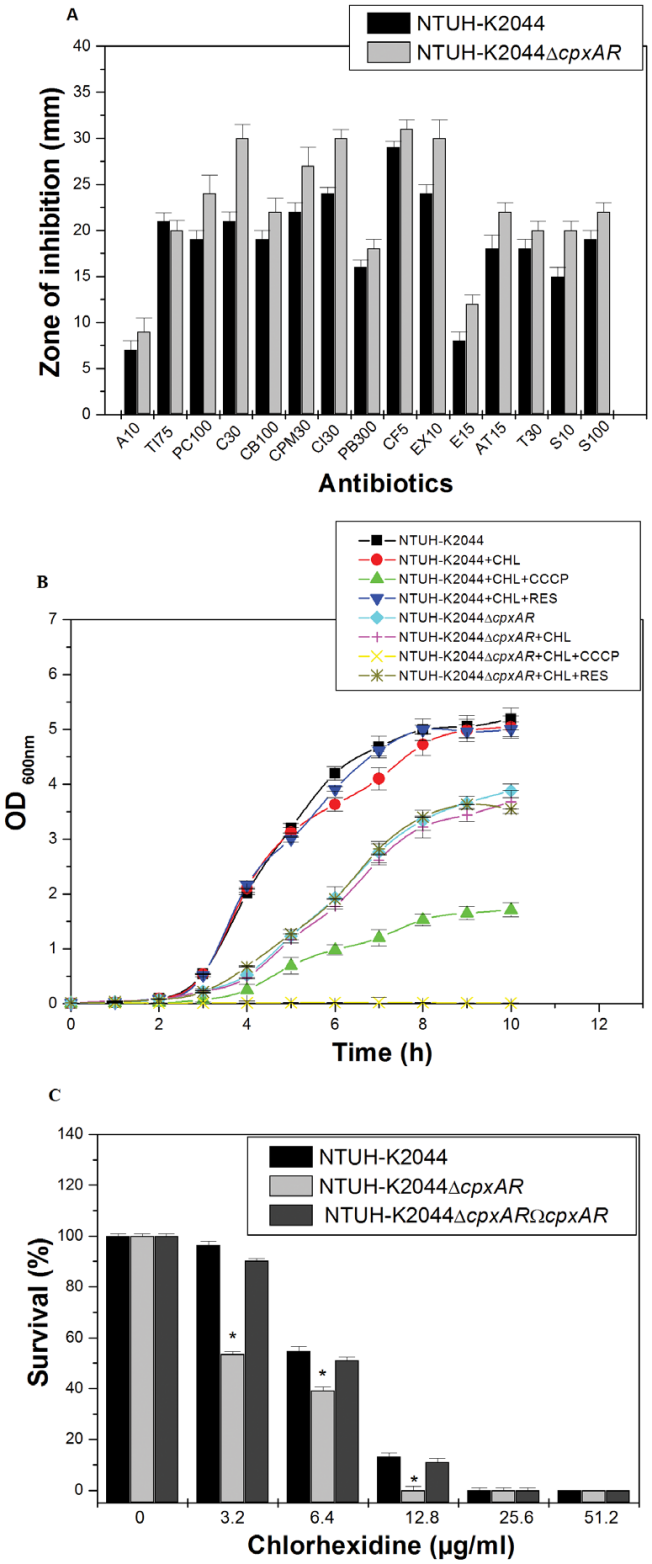
Antibiotic susceptibility testing, *in vitro* growth inactivation and chlorhexidine challenge assay with NTUH-K2044, NTUH-K2044Δ*cpxAR*and NTUH-K2044Δ*cpxAR*Ω*cpxAR*. **A**) The Kirby Bauer disc diffusion assay was performed with different antibiotics (A10, Ti75, PC100, C30, CB100, CPM30, CI30, PB300, CF5, EX10, E15, AT15, T30, S10 and S100) using commercial discs. Data for representative drugs from each class have been shown here. B) Growth inactivation assays using chloramphenicol (0.005 µg/ml). The efflux pump inhibitors CCCP and reserpine were used at a concentration of 10 µg/ml in the experiment. Abbreviations: CHL = chloramphenicol; RES = reserpine; CCCP = cyanide m-chloro phenyl hydrazone. C) Sensitivity towards disinfectant chlorhexidine was monitored by using the agar dilution assay for different concentrations (3.2 µg/ml, 6.4 µg/ml, 12.8 µg/ml, 25.6 µg/ml, 51.2 µg/ml) for the wild-type strain, and *cpxAR* mutant. The datas are the means of measurements made in triplicate and performed three times. *, Significant difference (P<0.05, Student t test).

To evaluate the role of *cpxAR*, antibiotic susceptibilities of NTUH-K2044 and NTUH-K2044Δ *cpxAR* was monitored. The results of disc diffusion assay displayed that upon deleting the cell envelope response system the bacterial cells significantly displayed sensitivity to β-lactam group of antibiotics (imipenem, cefepime, ceftriaxone, ceftazidime, cefotaxime) and chloramphenicol (Figure 5A).

The precise minimum inhibitory concentration (MIC) was further evaluated by following the guidelines of CLSI by E-test. The MIC for *K. pneumoniae* NTUH-K2044 for the different antibiotics was cefepime (2.048 µg/ml), ceftriaxone (0.016 µg/ml), ceftazidime (0.256 µg/ml), cefotaxime (0.256 µg/ml), and chloramphenicol (0.1 µg/ml) respectively.

The MIC for *K. pneumoniae* NTUH-K2044Δ*cpxAR* {fall in MIC fold in brackets} for the same line of drugs were cefepime (0.512 µg/ml, {4 fold}), ceftriaxone (0.016 µg/ml, {1 fold}), ceftazidime (0.128 µg/ml, {2 fold}), cefotaxime (0.064 µg/ml, {4 fold}), and chloramphenicol (0.01 µg/ml, {10 fold}) respectively (Table 2).

**Table 2:** Determination of MIC for wild type (NTUH-K2044), mutant (NTUH-K2044Δ*cpxAR*) and complemented (NTUH-K2044Δ*cpxARΩcpxAR*) strains.

Antibiotics	NTUH-K2044	NTUH-K2044Δ*cpxAR*	Fold change^a^	NTUH-K2044Δ*cpxAR* Ω*cpxAR*
Cefepime	2.048	0.512	4	>1.536
Cefotaxime	0.256	0.064	4	0.256
Ceftazidime	0.256	0.128	2	0.256
Ceftriaxone	0.016	0.016	1	0.016
Chloramphenicol	0.1	0.01	10	100
Erythromycin	0.01	0.01	1	0.01
Nalidixic acid	0.01	0.01	1	0.01
Polymyxin	0.1	0.1	1	0.1
Streptomycin	0.1	0.1	1	0.1

E-strips were used to determine the precise MIC for different group of antibiotics such as amikacin, ampicillin, cefepime, cefotaxime, ceftazidime, ceftriaxone, chloramphenicol, ciprofloxacin, erythromycin, kanamycin, nalidixic acid, ofloxacin, polymyxin, rifampicin, tetracycline, tobramycin, trimethoprim, and vancomycin following the CLSI guidelines.

a Fold change is the ratio of MICs for NTUH-K2044 and NTUH-K2044Δ *cpxA*.

### CpxAR influences drug efflux to confer antibiotic resistance

To decipher whether *cpxAR* confers antibiotic resistance by affecting drug efflux, screening for a potential efflux phenotype was accomplished by determining the growing ability of NTUH-K2044 and NTUH-K2044Δ*cpxAR* in the presence of chloramphenicol and CCCP or reserpine (10 µg/ml) as described in methods section [13]. The growth rate of NTUH-K2044Δ*cpxAR* in the presence of 0.005 µg/ml chloramphenicol (MIC being 0.01 µg/ml) was 2 fold lower than that of NTUH-K2044 [wild type/Δ*cpxAR* mutant, P = 0.00659].

Conversely, both wild type and Δ*cpxAR* mutant exhibited stunted growth in the presence of chloramphenicol and protonophore CCCP (Figure 5B). In independent experiments, growth remained unaltered on the addition of reserpine. Overall, preliminary findings clearly revealed that *cpxAR* utilises drug efflux as one of the mechanism to confer resistance against antimicrobial compounds such as chloramphenicol.

### CpxAR confers cross resistance to disinfectants

*K. pneumoniae* is a nosocomial pathogen and has an ability to stay in abiotic surfaces for long [14]; therefore we tested the susceptibilities of NTUH-K2044 and NTUH-K2044Δ*cpxAR* towards different concentrations of popularly used disinfectant chlorhexidine (Figure 5C) and benzalkonium chloride (data not shown) in hospitals.

The percent survival of NTUH-K2044Δ*cpxAR* was reduced to 50% upon the lowest exposure of chlorhexidine [wild type/Δ*cpxAR* mutant, P = 0.014], indicating that *cpxAR* has a contributory role to mediate disinfectant resistance in this nosocomial pathogen.

### Outer membrane profile of *cpxAR* deletion mutant in *K. pneumoniae*

**Figure 6:**
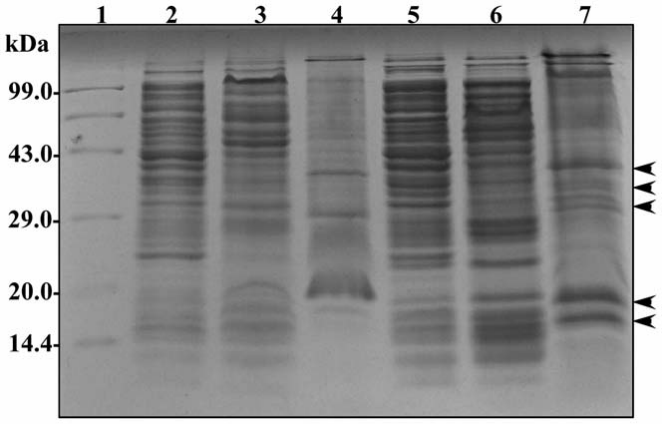
Comparison of membrane protein profiles of NTUH-K2044and NTUH-K2044Δ*cpxAR*. Membrane protein profiles were compared between the wild-type strain, and *cpxAR* mutant. Total protein lysate of wild-type strain (lane 2), outer membrane fractions (lane 3), inner membrane fractions (lane 4), followed by total protein lysate of *cpxAR* mutant (lane 5), outer membrane fractions (lane 6), inner membrane fractions (lane 7). Equal protein concentrations were separated by SDS-PAGE with a 5% stacking gel and a 12% separating gel and stained with coomassie brilliant blue. Lane 1 has molecular weight standards. The over expressed bands in outer membrane fractions of *cpxAR* mutant are shown by arrow heads.

The cell envelope is the prime line for most outside stress conditions that may modify envelope components and thus bring an extra cytoplasmic stress response. In our present study, we found that CpxAR contribute to antibiotic resistance more precisely towards cefepime and chloramphenicol resistance. A reduction in the permeation of antibiotics is generally related to a decrease in porin expression or an alteration in the porin structure [15]. To get an insight, we evaluated the membrane profiles of *cpxAR* mutant and the wild type. Analysis revealed alterations in both inner and outer membrane fractions of wild type and mutant, however it was intriguing to note the presence of over expressed bands in the outer membrane fractions of *cpxAR* mutant in varying sizes ∼30 kDa, ∼22 kDa and ∼16 kDa respectively (Figure 6).

### CpxR binds to the promoter region of OmpC^KP^ in *K. pneumoniae*

The classical porins OmpF and OmpC are major constituents of the *E. coli* outer membrane and account for approximately 2% of the total protein content of the cell [5]. These proteins allow for the passive diffusion of solutes across the outer membrane. Many environmental factors have been identified that alter OmpF and OmpC expression, including osmolarity, temperature, pH, nutrient availability, and various toxins [5].

**Figure 7:**
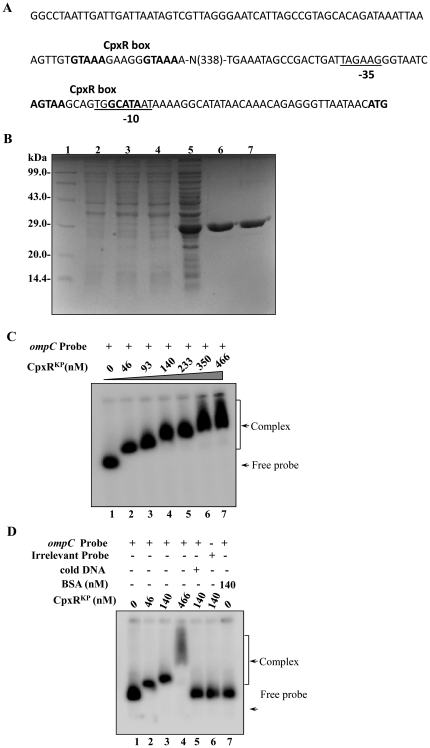
Cloning, expression and purification of CpxR and gel retardation assays using radio labeled *ompC* ^KP^ promoter. **A**) Promoter region analysis of *ompC* ^KP^. The numbers in brackets represent the distance from the transcription start site. The −35 and −10 region in the promoter is underlined. CpxR box has been shown in bold. B) SDS-PAGE profile of pET-CpxR^KP^. Lane 1: medium size marker, Lane 2: pET28C/BL21DE3 uninduced, Lane 3: pET28C/BL21DE3 induced, Lane 4: pET-CpxR^KP^/BL21DE3 uninduced, Lane 5: pET-CpxR^KP^/BL21DE3 induced, purified CpxR^KP^-fractions E1 and E2 (lanes 6–7) respectively. Protein samples after induction were subjected to SDS/PAGE (15% gel) followed by coomassie brilliant blue staining. C) Gel shift assays demonstrating the binding of CpxR to promoter of outer membrane protein OmpC in *K. pneumoniae* in a concentration dependent manner. Lane 1 (shows free probe), lanes 2–7 with increasing concentrations of CpxR protein (46 nM to 466 nM) respectively. Slower moving bound complexes and free probe has been indicated by arrows respectively. The gels are representative of at least three independent experiments. D) Gel shift assays demonstrating the sequence-specific binding of CpxR to *ompC* using different controls as in lane 1 (shows free probe), lanes 2–4 (labeled *ompC* promoter with increasing amount (46 nM, 140 nM and 466 nM) of CpxR), lane 5 (labeled *ompC* promoter and CpxR with specific competitive inhibitor: 10 fold excess of unlabeled *ompC* promoter), lane 6 (labeled non-specific DNA: promoter of *gyrA* and CpxR, 140 nM), lane 7 (labeled *ompC* promoter with non-specific protein: BSA, 140 nM) respectively. The gels are representative of at least three independent experiments.

The binding site of the RR CpxR in the upstream regulatory region of its target genes has been identified before in *E. coli* and in *S. sonnei* [6]. According to these reports, CpxR has a conserved recognition site that contains the sequence GTAAA. A previous study indicated that strong CpxR regulation in *E. coli* can be correlated with the presence of this motif within 100 bp in the 5′ direction from the transcriptional start site [5], [6]. The DNA binding feature of CpxR prompted us to analyse the promoter regions of outer membrane proteins for the existence of putative CpxR binding sites. Interestingly analysis revealed the presence of a conserved putative CpxR binding site spanning region between 34 to 50 bp from the first methionine of OmpC^KP^ (Figure 7A). The OmpC^KP^ (homologue of *E. coli* OmpC) is found in the genome of *K. pneumoniae* KP1_3869, a 1098 bp gene that encodes a polypeptide of 365aa (40 kDa).

To define the possible interaction of CpxR^KP^ to the promoter of OmpC^KP^, we tested whether CpxR^KP^ directly interacts with its promoter region. For that we first cloned and expressed the *cpxR* gene. The *cpxR* gene from *K. pneumoniae* was PCR amplified, cloned into pET-28c and after transformation in *E. coli* strain BL21(DE3), expression of the His-tagged protein (CpxR^KP^) was monitored following IPTG induction. Cell lysates following purification on a Ni-NTA column, where resolved by SDS/PAGE yielded an expected induced band of ∼24 kDa (Figure 7B).

Thereafter, gel shift assays were performed using the ^32^P-labeled *ompC* ^KP^ promoter fragment and CpxR. The protein-DNA complexes after incubation in reaction buffer were resolved on 5% PAGE gel and analysis revealed a clear retardation which was proportional to the protein concentration as shown in the Figure 7C. Use of various controls such as competitor inhibitor (specific and non-specific: poly (dI-dC)), irrelevant protein as shown Figure 7-D clearly demonstrated the specific DNA binding ability of CpxR^KP^ to promoter region of outer membrane protein *ompC* ^KP^in *K. pneumoniae*.

### Expression analysis of the efflux genes in *K.pneumoniae*

**Figure 8:**
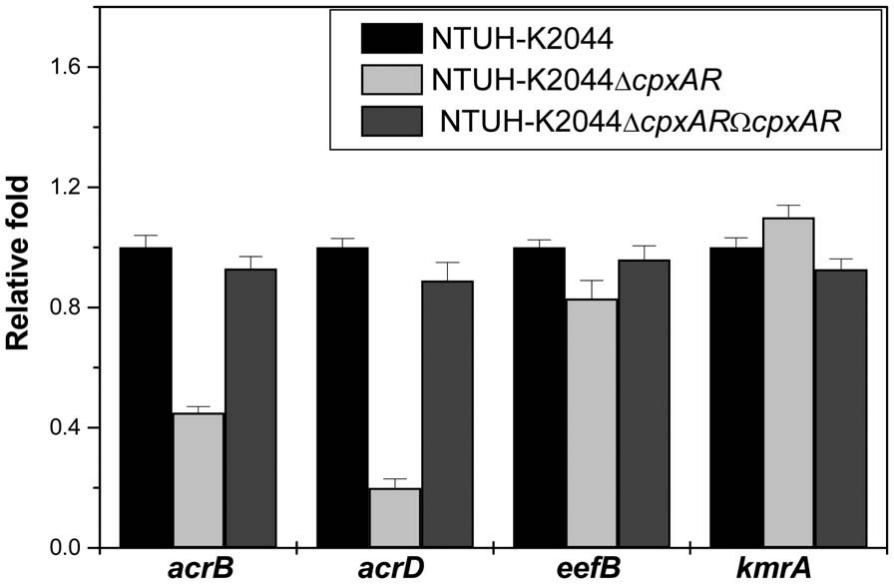
Quantification of the transcript levels of multidrug transporter genes that belong to RND-type and MFS-type efflux pumps. Relative transcriptional levels of *acrB, acrD, eefB, kmrA* in NTUH-K2044Δ*cpxAR* and NTUH-K2044Δ*cpxAR*Ω*cpxAR* strains determined using real time RT-PCR is showed in comparison with wild type. The wild type expression level is represented as one fold. Each bar represents the average value of three independent experiments. Error bars are standard deviations.

Quantitative real-time RT-PCR (qRT-PCR) was used to examine expression of the efflux transporter genes in wild-type, *cpxAR* mutant, and *cpxAR* complemented strains. Compared to the wild-type strain, expression of resistance-nodulation-cell division (RND) efflux pump such as *acrB, acrD* and *eefB* genes were decreased by 3 fold, 5 fold and 2 fold respectively in the *cpxAR* mutant (For *acrB*, Δ*cpxAR* and wild type: *P*<0.0001, *acrD*, Δ*cpxAR* and wild type: *P*<0.0001, and *eefB*, Δ*cpxAR* and wild type: *P*<0.0001, Student's *t* test.), while there was a marginal increase in expression of major facilitator (MFS) type efflux pump *kmrA* compared to wild type (*P* values<0.0001) (Figure 8). Complementation of the *cpxAR* mutation almost restored expression of all the tested genes (*P* values<0.0001) (Figure 8). These results provide evidence for the additional regulatory role of Cpx system on MDR efflux pumps.

## Discussion

Bacteria have numerous different systems for sensing their environment and to respond with alterations in gene expression. Given the significance of the integrity of the cell envelope to bacterial survival, it is known that five different systems which respond to stresses in the cell envelope have been explored. Among these, the CpxAR TCS is conceivably the best characterized [2]. At least two important functions have been ascribed to the Cpx system in enteric bacteria; these include regulating factors that deal with misfolded proteins in the periplasmic space and affecting expression of surface components that mediate attachment to some surfaces. It has also been suggested that the Cpx signaling pathway may play a role in signaling *E. coli* cells present in biofilms to stop making biofilm-related adhesins [16]. The signals that activate the Cpx system in *E. coli* are diverse and include alkaline pH, overexpression of certain proteins, interaction with abiotic surfaces, and others. The Cpx regulon in *E. coli* has been described as involving 34 operons and at least 50 genes [17]. In this investigation the unprecedented area *i.e.* its direct involvement in drug resistance has been decoded in *K. pneumoniae*. The recently sequenced genomic data of *K. pneumoniae* NTUH-K2044, encoding 4,992 proteins reveals the presence of CpxAR operon in its genome [10]. The operon was disrupted and its effect on general physiology of the pleomorphic bacillus was studied.

The mucoid slimy nature of cells is indicative of an overproduction of a capsule like polysaccharide in *K. pneumoniae*, but in our study no significant difference in capsule synthesis between the *K. pneumoniae* wild-type strain and its *cpxAR* mutant was detected. It would be worthy to state here that other factors such as the production of exopolysaccharides, pilus synthesis, lipopolysaccharide composition, or the expression of auto transporter proteins, also are responsible for capsule synthesis [18], thus mere deletion of *cpxAR* might not be sufficient to see a loss in capsule production.

*K. pneumoniae* is an opportunistic pathogen responsible for many nosocomial infections. Multidrug resistant *K. pneumoniae* isolates are frequently isolated at an increased frequency, which therefore leads to a therapeutic impasse [19]. The reservoir for *K. pneumoniae* strain is the GI tract of patients, and GI colonization depends on the ability of the bacteria to adhere to mucosal surfaces, to form biofilm within the mucus layer, and to resist the specific stresses encountered in the GI tract [9]. Epidemiological studies have shown that, whatever the infection site, the first stage in nosocomial infections due to *K. pneumoniae* consists of the colonization in the patient's GI tract [20]. This pathogen therefore has to sense and respond to numerous different environments in order to survive and, consequently, to persist in the GI tract of the host. The first major barrier encountered following oral consumption is stomach acidity. The bacteria then enter the small intestine, where they encounter stresses associated with volatile fatty acids, variations in pH and osmolarity, and competition with endogenous flora [12].

The behaviour of the wild-type strain and the *cpxAR* deficient mutants was therefore investigated under some of these hostile conditions to further understand how *cpxAR* interacts in the GI tract. In the presence of osmotic and bile challenges, *cpxAR* mutant exhibited two to three fold lower survival capabilities than the wild type, and its level of growth in physiological pH was severely impaired. It is likely, therefore, that the *cpxAR* factor controls functions required for survival of bacteria in the upper parts of the GI, where they encounter both high osmolarity and bile salts in a microaerobic environment.

Reactive oxygen species, including hydrogen peroxide (H_2_O_2_), superoxide, and hydroxyl radical are toxic to cells due to their ability to damage DNA and especially proteins containing iron-sulfur clusters or sulfur atoms [21]. In bacteria, many transcription factors have been found to sense the presence of reactive oxygen species and induce antioxidant system. Evidence for the involvement of *cpxAR* system in oxidative stress tolerance has been demonstrated in this study.

Antimicrobial therapy for *K. pneumoniae* is often ineffective as members of the *Klebsiella spp*., are highly resistant to most clinically relevant antimicrobial agents including disinfectants. MDR in *K. pneumoniae* isolates is defined as resistance to all of the agents belonging to at least two of three classes of antibiotics, such as quinolones, aminoglycosides, and β-lactam agents. Yamaguchi *et al*, demonstrated the role of TCS to β-lactam resistance in *E. coli*. Thirteen RRs present in *E. coli* genome including *cpxR* when over reproduced in drug hypersensitive heterologous host, conferred increased β-lactam resistance [22]. In this study we studied the role of *cpxAR* in antibiotic and disinfectant resistance. Deletion of *cpxAR* made cells sensitive to β-lactam drugs such as cefepime, cefotaxime, ceftazidime and chloramphenicol which possibly are the preferred substrates of the resistance determinants for *e.g.* efflux pumps perhaps regulated by the Cpx regulon.

In spite of regular preventive surface disinfection in clinical settings, inanimate surfaces have often been described as the source for the persistence of multidrug resistant *K. pneumoniae.* This study provides primary evidence for the participation of *cpxAR* TCS in mediating disinfectant resistance.

Drug efflux represents an important protection mechanism in bacteria to withstand antibiotics and environmental toxic substances [13]. Efflux genes constitute 6–18% of all transporters in bacterial genomes [12]. Particularly interesting among mediators of MDR in *K. pneumoniae* are the efflux pumps belonging to MFS, RND, ATP binding cassette, small multidrug resistance, multidrug and toxic compound extrusion families [23]. In *K. pneumoniae* NTUH-K2044, deletion of *cpxAR* resulted in loss of drug efflux capacity. The *cpxAR* deletion reduced the expression levels of efflux genes such as *acrB, acrD and eefB* in mutant when compared to wild type which indicates that possibly CpxR has a role in modulating the expression of MDR efflux pumps.

Classical efflux pump comprises of an outer membrane protein that serves as a channel to regulate the exchange of extra and intra cellular substances which also includes antibiotics, detergents, dyes, organic solvents and bile acids [12]. It was interesting to observe that the outer membrane protein profile of NTUH-K2044Δ*cpxAR* had over expressed protein bands at ∼30 kDa, ∼22 kDa and ∼16 kDa.

Recently, it has been reported that double deletion of OmpK35 (homolog of OmpF in *E. coli*) and OmpK36 (homolog of OmpC in *E. coli*) altered the MIC's of β-lactams class of drugs in *K. pneumoniae* [7]. Previous study has shown that the expression levels of outer membrane proteins STM1530 and OmpD, were influenced by the *cpxAR* TCS and it played important roles in mediating ceftriaxone resistance of *S.* Typhimurium [24].

The notable differences in membrane profile prompted us to investigate the presence of CpxR binding sites in functionally characterized porins such as OmpC homolog (OmpK36) in *K. pneumoniae*. Evidence for the CpxR protein binding on the promoter fragments of OmpC^KP^ has been shown for the first time which well corroborate with the reports documented previously in other Gram negative bacteria.

One striking feature of the predicted protein complement of *K. pneumoniae* is the presence of a disproportionately large number of regulatory proteins [10]. Besides the presence of TCS there exists the presence of eukaryotic-type Ser/Thr kinase known as one component system in bacterial genomes. Recently, our group has identified the presence of one component system homologue in *K. pneumoniae* (data to be communicated). Thus it is not only important to decode the regulatory cascade of the TCS but it is also imperative to understand the correlation between the one component system and TCS to get an overview of global signaling networks in clinically significant pathogens.

Thus, characterizing the functions of CpxAR operon marks just the beginning by in itself. In summary, this study provides preliminary experimental evidence for the participation of cell envelope stress response system CpxAR in mediating resistance against GI stresses, antibiotics and disinfectants in *K. pneumoniae* NTUH-K2044; hyper virulent K1 serotype for the very first time.

## Materials and Methods

### Bacterial strains, plasmids and media

*K. pneumoniae* NTUH-K2044 (a strain that resulted in pyogenic liver abscess in a 66 year old patient) was kindly provided by Dr. Jin Town Wang of the National Taiwan University Hospital, Taipei, Taiwan [25]. *E. coli* S17-1λ pir which carries the F plasmid and encodes π protein essential for replication of pUT-Km was used for cloning experiments. pUT-Km was used to create insertion-duplication mutations by homologous recombination. Bacteria cultures were grown in Luria-Bertani (LB) broth or on LB agar (Difco, Becton-Dickinson, Sparks, MD) at 37°C with constant shaking (220 rpm) and supplemented with Kanamycin (100 µg/ml) where required. The strains were harvested and stored at −80°C before use.

### DNA methods

Restriction digestion, ligation, transformation, and agarose gel electrophoresis were done according to standard protocols. Plasmids were prepared from *E. coli* using a QIAprep Spin miniprep kit from Qiagen according to the manufacturer's protocol. Mobilization of plasmids into *K. pneumoniae* cells was performed as previously described [12]. Genomic DNA of *K. pneumoniae* was extracted using the Gene Aid DNA purification kit according to the manufacturer's instructions. DNA fragments used for cloning were extracted from agarose gels using a QIA quick gel extraction kit from Qiagen. PCR products were purified using a QIA quick PCR purification kit (Qiagen) and, when cloned, sequenced to confirm the correct sequences (Applied Biosystems). Primers used in the present study were custom-synthesized (Eurofins MWG operons, Germany).

### Construction of the *cpxAR* deletion mutant in *K. pneumoniae* strain NTUH-K2044

The MisT2 database (http://mistdb.com) shows the presence of >466 signaling proteins in the 5,472,672 bp (GC content: 57.4%) genome sequence of the K1 serotype (Accession No: AP006725.1). The CpxAR operon is located starting from nucleotides 76799 bps to 78867 bps (*cpxA*: 1373 bp, 457aa and *cpxR*: 698 bp, 232aa) in the genome sequence of *K. pneumoniae* NTUH-K2044. To construct *cpxAR* knock out, a 700 bp internal fragment encompassing *cpxA* and *cpxR* of the operon was amplified by PCR using Δ*cpxA/cpxR*-F and Δ*cpxA/cpxR*-R primer from its genomic DNA (Table 3). The PCR product was ligated into an *EcoR*I digested plasmid pUT-Km which was blunted by klenow reaction (pUT-Km is a pUTKm1 derived plasmid, with miniTn5 excised by EcoRI, tnp excised by SalI, and bla removed by ApaLI, then with an insertion of kanamycin resistance cassette from pUC4K into PstI site) that contained the kanamycin resistance gene, transformed into *E. coli* S17-1λ pir and the resulting recombinant plasmid harbouring the internal fragment of *cpxAR* was designated as pUT-Km/GR. The plasmid pUT-Km/GR was mobilized into recipient *K. pneumoniae* NTUH-K2044 from donor *E. coli* S17-1λ pir.

**Table 3:** Primers used in this study.

Primer name	Primer sequences (5′-3′)
Δ*cpxA/cpxR*-F	GGAACGAAGTGTTGGACAATGCGGCGTTTG
Δ*cpxA/cpxR*-R	TTATCGATGGCGCGAAACAAACGAC
Primer NT	GCGCATCACATACTCCCAAAACGTTTGTGT
Primer CT	CCCATATTAAGTATTCCCGGGGATGCTCC
*cpxR*-F	GCGTCATATGAATAAAATCCTGTTAGTTGATGATG
*cpxR*-R	CTCAGGATCCTCATGAAGCGGAAACCATCAGATAC
prom *ompC*-F	GGCCTAATTGATTGATTAATAGTCGTTAGGGAAT
prom *ompC*-R	GTTATTAACCCTCTGTTTGTTATATGCCTTTTAT
*eefB*nt	TTCTCGGTAACCATTATCTCGGCGATGATG
*eefB*ct	TTATCTCCCCCTGGTCCTCCACCGG
*acrB*nt	AAGAGCACGCACCATTACACCGACAG
*acrB*ct	TTCCTCACCCGGACGCTGGCTCCAGTC
*acrD*nt	GATCCTGCTGGTCTTCCTCGTGAT
*acrD*ct	GCCCTGAATTTGTCCCATCGATTT
*kmrA*nt	TGCTGGAGCATTTCTACTGGGGGTCGGTGT
*kmrA*ct	TGCAGCTCCTGGGCCATCAGTAGCTCAAAA
16sF (*rrsE*)	CAGCCACACTGGAACTGAGA
16sR (*rrsE*)	GTTAGCCGGTGCTTCTTCTG

Briefly, *K. pneumoniae* was inoculated into 10 ml LB and was incubated for 2–3 h till OD_600 nm_ reaches 0.2. For matings, recipient and donor culture were mixed in a ratio of 1∶2 respectively, pelleted and spotted onto the centre of an LB agar plate. After 3 h of growth at 37°C the cells were plated on *Klebsiella* selective agar (HiMedia HiCrome *Klebsiella* Selective Agar Base cat# M1573; *Klebsiella* Selective Supplement cat# FD225) containing Kanamycin 100 µg/ml and 5 µg/ml chlorhexidine to select for colonies. It is expected that colonies that appear on the selective plate would be transconjugants that resulted from one DNA exchange event in which the whole suicidal plasmid gets incorporated in the *K. pneumoniae* genome. The disruption at *cpxAR* operon was confirmed with selected transconjugant by PCR and DNA sequencing using gene specific and genome flanking primers and deleted mutant was denoted as NTUH-K2044Δ*cpxAR*.

Intact *cpxAR* genes were amplified along with its promoter using primer NT (binds −155 bases upstream of *cpxR*) and primer CT (binds +64 bases downstream of *cpxA*) and cloned into a pCRIITOPO-CAT plasmid (Table 3). The selected recombinant plasmid harbouring the intact *cpxAR* operon was transformed into the *cpxAR* isogenic mutant strain by electroporation. The complementation strains were selected on LB agar plates supplemented with 100 µg/mL kanamycin and 100 µg/mL chloramphenicol and transcomplemented strain was designated as NTUH-K2044Δ*cpxAR*Ω*cpxAR*.

### String and Precipitation test for Hypermucoviscosity

The NTUH-K2044 and NTUH-K2044Δ*cpxAR* was streaked onto LB agar plates and incubated at 37°C overnight. A standard bacteriologic loop was used to stretch a mucoviscous string from the colony. Hypermucoviscosity was defined by the formation of viscous strings >5 mm in length when a loop was used to stretch the colony on agar plate which was considered the positive string test [19]. The strains to be tested were cultured overnight in LB broth at 37°C and subjected to centrifugation at 1,000×*g* for 5 min to check reduction in mucoidy.

For exopolysaccharide analysis [26], cells were grown to late log phase in shaking culture and stained with crystal violet followed by treatment with 20% copper sulphate solution (Anthony's capsule staining methodology). Samples were visualized using an Olympus microscope work station. Capsular polysaccharides were extracted from overnight bacterial suspensions adjusted to ∼10^8^ cells per ml with Zwittergent 3–14 detergent. The amount of uronic acid was then measured according to the method described previously [27]. Each experiment was performed in triplicate.

### Scanning electron microscopy

Overnight cultures were fixed after harvesting; cells were washed three times with ice-cold NaCl/Pi. The cells were then resuspended in NaCl/Pi, adhered to cover slips that had been coated with 0.1% poly (l-lysine). Adherent cells were washed with NaCl/Pi and then dehydrated using an ascending series of ethanol incubations. Finally, cells on covers lips were infiltrated with t-butyl alcohol and freeze-dried in a lyophilizer. Dried samples were sputter-coated with gold/palladium and then observed under a scanning electron microscope [28].

### *In vitro* growth curves

To examine bacterial growth *in vitro*, overnight cultures were diluted 1∶100 and subcultured for 10 h. The growth kinetics was monitored with LB at different pH (3, 6, 8 and 12). The growth inhibition assay was performed as described previously [13]. The efflux pump inhibitors (10 µg/ml) used in this study was carbonyl cyanide 3-chlorophenylhydrazone (CCCP) and reserpine (Sigma, St. Louis, MO). CCCP is an extremely effective proton motive force inhibitor and used in this study as an active efflux pump blocker. Efflux pump inhibitors had no intrinsic antibacterial activity against wild type strain at the concentration used in the experiments.

### Osmotic, bile, chlorhexidine challenge assays

Various stress assays were performed as described previously [12]. Briefly, *K. pneumoniae* NTUH-K2044 and NTUH-K2044Δ*cpxAR* were grown to mid-exponential phase, cultures were spread plated onto LB agar plates containing different concentrations of NaCl (0.075 M, 0.15 M, 0.25 M, 0.5 M, 0.75 M, 1.0 and 2.0 M), bile (0.2%, 0.5%, 0.75%, 1.0%, and 2.0%) and chlorhexidine (3.2 µg/ml, 6.4 µg/ml, 12.8 µg/ml, 25.6 µg/ml, 51.2 µg/ml) respectively. The results are expressed as the ratio of the number of colony forming units obtained from LB cultures containing different concentrations of NaCl, bile and chlorhexidine to the number of colony forming units obtained from control cultures (LB agar alone). These experiments were performed at least three times.

### Oxidative stress sensitivity assay

In this susceptibility test, small Whatman 3 MM paper disks (6 mm) was impregnated with different amount of H_2_O_2_ (10 µl of 3%, 10% and 30%) and later air dried as reported before [27]. The *K. pneumoniae* NTUH-K2044 and NTUH-K2044Δ*cpxAR* were grown to the mid-log phase (OD_600 nm_ 0.2) and was uniformly spread over an LB agar plate. Next, filter paper disks impregnated with specific concentrations of H_2_O_2_ was placed at the centre on to the agar surface. The culture was then incubated at 37°C for 12–24 hours. The diameter of a zone of inhibition was measured (in millimeters) which is a qualitative measure of the inhibitory activity of a compound. The data represents the distances from the edge of the disks to the end of the clear zone, where growth begins. Each experiment was repeated at least three times.

### Antibiotic susceptibility testing

Strains in this study were examined for resistance to nalidixic acid: NA30 (30 µg/ml), colistin: CL30 (10 µg/ml), enrofloxacin: EX10 (10 µg/ml), polymyxin B: PB300 (300 µg/ml), ciprofloxacin: CF5 (5 µg/ml), azithromycin: AT15 (15 µg/ml), erythromycin: E15 (15 µg/ml), tetracycline: T30 (30 µg/ml), rifampicin: R5 (5 µg/ml), trimethoprim: TR5 (5 µg/ml), kanamycin: K30 (30 µg/ml), streptomycin: S10 (10 µg/ml), tobramycin: TB10 (10 µg/ml), clindamycin: CD2 (2 µg/ml), spectinomycin: S100 (100 µg/ml), imipenem: I10 (10 µg/ml), ampicillin: A10 (10 µg/ml), ertapenem: ETP10 (10 µg/ml), piperacillin: PC100 (100 µg/ml), ticarcillin: TI75 (75 µg/ml), ceftazidime: CA30 (30 µg/ml), chloramphenicol: C30 (30 µg/ml), ceftriaxone: CI30 (30 µg/ml), cefepime: CPM30 (30 µg/ml) and carbencillin: CB100 (100 µg/ml) by using commercial discs (Hi Media, Mumbai, India) as described previously according to the interpretation criteria recommended by Clinical and Laboratory Standards Institute CLSI [29].

MIC of antibiotics was tested using E-strips. Interpretation was done as per the criteria approved by CLSI [29]. *E. coli* ATCC 25922 was used as a reference strain (control) as recommended.

### Outer membrane proteins preparation

Outer membrane proteins were purified by the method as described previously [30]. Cells were harvested by centrifugation (5,000× g for 15 min) and were suspended in 50 mM Tris-HCl buffer (pH 7.4) (Tris buffer) containing 5 mM phenylmethylsulfonyl fluoride and sonicated for 15 mins. The crushed material was treated with DNase and RNase (each at 100 µg/ml), and the unbroken cells were removed by centrifugation (10,000× g for 10 min). The crude envelope fraction was collected from the supernatant by centrifugation at 105,000× g for 1 h at 4°C. The pellet containing the crude envelope fraction was treated with 0.5% (wt/vol) Sarkosyl (Sigma) solution to selectively solubilise the inner membrane part. The insoluble outer membrane fraction was recovered as pellet by centrifugation at 105,000× g for 1 h at 4°C. The pellet was washed and stored at −20°C until used. Protein contents of membrane preparations were determined by the method of bicinchoninic acid (BCA) method (Pierce BCA protein assay kit, cat# 23225) with bovine serum albumin (BSA) (Sigma) as standard.

### Gene cloning, expression, purification and electrophoretic mobility shift assays

The DNA-binding transcriptional regulator gene *cpxR* was amplified using gene specific primers, *cpxR-F* and *cpxR-R* has NdeI and BamHI site of the pET28C vector to generate an N-terminal His_6_-CpxR fusion protein. All clones were confirmed by sequencing and transformed into *E. coli* BL21 (DE3). After induction with 0.2 mM isopropyl 1-thio-β-d-galactopyranoside, CpxR protein was purified through Qiagen Ni^2+^nitrilotriacetic acid columns. The protein was dialysed using Tris buffer pH 8.0.

The ability of purified recombinant CpxR to bind *ompC* promoter was examined by using the electrophoretic mobility shift assay (EMSA). The *ompC* promoter region was amplified using prom *ompC*-F and prom *ompC*-R primers (Table 3) and subjected to EMSA with purified CpxR protein. Briefly, end-labelled (using [γ-^32^P] ATP) PCR products were incubated with increasing concentrations (in a range of 40 nM to 500 nM) of CpxR in binding buffer (10 mM Tris-HCl, pH 8.0, 2 mM EDTA, 0.5 mM DTT, 50 mM NaCl, 10% glycerol, and 1 µg of poly(dI·dC). The complexes were run on 5% native polyacrylamide gel electrophoresis (PAGE) gels for 2 h. The gel was then dried and exposed to the phosphor screen for image analysis. To confirm that the interaction between CpxR and the promoter region of *ompC* was specific, competition experiments with bovine serum albumin (BSA) as a negative control (non competitive) and with 10 fold excess of cold promoter (competitive) were also performed.

### RNA isolation and real-time reverse transcription PCR (RT-PCR)

Total RNA was extracted from the log-phase cultures of *K. pneumoniae* NTUH-K2044 wild-type and *cpxAR* mutant using the RNeasy Mini Kit (Qiagen) according to the manufacturer's instructions. Total RNA was digested with DNase I to ensure the removal of contaminating genomic DNA prior to cDNA synthesis. Aliquots of 500 ng of DNase treated total RNA served as template for complementary DNA (cDNA) synthesis using SuperScript III Reverse Transcriptase (Invitrogen). The cDNA samples were diluted 1∶10 and 2 µL was used per 25 µL quantitative PCR reaction for different efflux genes such as *acrD*: (KP1_4054: aminoglycoside efflux system), *kmrA* (KP1_2943: *smvA*; energy-dependent efflux protein for methyl violgen), *acrB* (KP1_1319: acriflavine resistance protein B), *eefB* homolog (KP1_5407: acridine efflux pump) were performed using gene specific primers (Table 3).

Gene expression levels were monitored by real time RT-PCR using Maxima SYBR Green qPCR master mix (Fermentas) in an iCycler thermal cycler (Bio-Rad) and the melting curve analysis were carried out to confirm amplification of a single product. Total RNA was isolated from at least two separately grown replicate cultures. All real time RT-PCR experiments were performed in triplicate, with 16sRNA used as an internal control.

### Bioinformatic analysis and Statistical analysis

The multiple sequence alignments were carried out using the Clustal program www.ebi.ac.uk Homology searches, similarities and identities analysis and conserved domain architecture analysis were performed using NCBI web server [31], Simple Modular Architecture Research Tool (SMART) www.smart.embl-heidelberg.de and NCBI conserved domain search. All data are presented as means ± the standard error of the mean. Plotting and calculation of the standard deviation was performed in Microsoft Excel. Statistical analysis was performed on crude data by using a paired Student t test. P values of <0.05 were considered significant.
